# Screening the Capacity of 34 Wetland Plant Species to Remove Heavy Metals from Water

**DOI:** 10.3390/ijerph17134623

**Published:** 2020-06-27

**Authors:** Maria Schück, Maria Greger

**Affiliations:** Department of Ecology, Environment and Plant Sciences, Stockholm University, 106 91 Stockholm, Sweden; maria.greger@su.se

**Keywords:** heavy metal removal, hydroponic, phytoremediation, wetland plants, water purification

## Abstract

Floating treatment wetlands (FTWs), consisting of vegetated rafts, may reduce heavy metal levels in polluted water, but the choice of plant species for efficient metal removal needs to be further investigated. We screened the capacity of 34 wetland plant species to remove metals dissolved in water to identify suitable species for FTWs. The plants were grown hydroponically for 5 days in a solution containing 1.2 µg Cd L^−1^, 68.5 µg Cu L^−1^, 78.4 µg Pb L^−1^, and 559 µg Zn L^−1^. Results show large variation in metal removal rate and capacity between the investigated species. The species with highest removal capacity could remove up to 52–94% of the metals already after 0.5 h of exposure and up to 98–100% of the metals after 5 days of exposure. Plant size contributed more to high removal capacity than did removal per unit of fine roots. *Carex pseudocyperus* and *C. riparia* were the most efficient and versatile species. The findings of this study should be considered as a starting point for further investigation of plant selection for improved water purification by FTWs.

## 1. Introduction

Wetland plant species are able to remove heavy metals from the surrounding environment, which can be utilized for purification of polluted waters. One such application is treatment of polluted stormwater, which is a growing problem due to increased traffic and heavy rains [1]. A common management of stormwater is stormwater ponds that collects and delays large water masses to prevent flooding. In these ponds, particulate heavy metals are effectively removed through sedimentation, but dissolved metals, proven to be more bioavailable than particulate metals, are insufficiently removed [2,3]. Heavy metals Cd, Cu, Pb, and Zn are the most problematic heavy metals in stormwater, since they are abundant, toxic, and risk to exceed threshold values in the outlet water [4].

The use of floating treatment wetlands (FTWs) has been proposed to increase the metal removal efficacy of stormwater ponds [5,6]. FTWs consist of rafts supporting emergent plants growing hydroponically, providing direct contact between the plant roots and the polluted water [7]. Compared with conventional constructed wetlands, FTWs require no additional land use because they can be placed in existing ponds. Moreover, FTWs tolerate large variation in water level, a common problem for plants in conventional wetlands. A few mesocosm and field studies of metal removal by FTWs have been conducted, most finding decreased concentration of both dissolved and particulate metals in the outlet water [2,6,8,9], in addition to increased metal concentration in the sediment [6,10] and in the plants [11,12,13].

Plants are necessary for the removal of metals by FTWs [2]. The root mass reduces the water velocity, allowing small particulate metals to settle out. Root activity and the microbial processes of root-living organisms may also change the chemical environment (e.g., pH and oxygenation), increasing the sedimentation or adsorption of metals [7]. Moreover, metals follow the water uptake into the plant, further decreasing the metal content in the water. Root activity and water uptake and, consequently, removal efficiency increase with increasing root biomass [14].

Various metals are taken up to different extents by plants. Zinc is generally accumulated to a higher extent than is Cu, followed by Cd and Pb [15,16]. These differences likely depend both on the selective uptake mechanisms of plants and on the availability of metals in water due to differences in retention time. In stormwater, the dissolved fractions easily available for uptake were found to be 5%, 38%, 53%, and 59% for Pb, Cu, Zn, and Cd, respectively [3].

The ability to accumulate metals is not the same in all species. Studies have found that species differ in their removal capacity due to differences in their uptake per unit biomass [17] or differences in plant size [16], resulting in differences in total removal per plant [12,18] or per vegetated area [2]. Furthermore, studies of plants in biofiltration systems have shown that bioaccumulation differs between families [15] and clades [19]. There is clearly potential to increase the efficacy of metal removal by FTW through identifying efficient plant species or families. Most studies of metal uptake, however, consider only one or a few species and one or a few metals. To our knowledge, no study has compared the ability of many plant species to remove several metals under identical conditions. Nevertheless, screening the performance of plants by exposing them to the same conditions allows an easy comparison between species [20]. Using a hydroponic setup for the comparison has been demonstrated as a useful approach to compare and identify species that performs well in field [21].

This study aims to identify species suitable for removing Cd, Cu, Zn, and Pb in FTWs by comparing 34 wetland plant species by quantifying the removal capacities and removal rates for these metals. The metals chosen represent abundant problematic heavy metals in stormwater, a potential target for phytoremediation with the investigated species.

## 2. Materials and Methods

### 2.1. Plant Materials

Specimens of 34 wetland plant species found in Swedish nature were used in the experiment (Table 1). They were either collected in the wild in the Stockholm area of Sweden, grown from seed in a climate chamber, or purchased from a seller of wild plants propagated from seeds collected in Sweden (Veg-Tech AB). The species naturally grow in freshwater, brackish water, or both (Table S1). Species that grow into large plants in the wild were generally represented in this study by large specimens; similarly, small-growing species were represented by small specimens.

### 2.2. Experimental Setup

The ability of species to remove heavy metals from water was tested in a hydroponic microcosm study in a greenhouse (13.5 h light with 92 ± 8 µmol PAR m^−2^ s^−1^, 60% relative humidity, 22 °C). The plant roots of each species were thoroughly rinsed of soil and debris with tap water at least two weeks prior to the start of the experiment. Thereafter, the plants were grown hydroponically in aerated modified Hoagland solution [22] in the greenhouse. At the start of the experiment, three similar and healthy-looking plants of each species were chosen. The plant roots were thoroughly rinsed again in deionized water to remove nutrient solution, and any dead plant material was carefully removed. Each plant was mildly shaken to remove excess water. The plant was attached to a plastic foam plate and placed in a 1-L acid-washed plastic beaker filled with 870 g of deionized water containing 1.2 µg Cd L^−1^, 68.5 µg Cu L^−1^, 78.4 µg Pb L^−1^, 559 µg Zn L^−1^, and 55.4 mg Cl L^−1^ added as CdCl_2_, CuCl_2_, PbCl_2_, ZnCl_2_, and NaCl. The pollutant concentrations were based on expected values in stormwater from highly travelled Swedish roads treated with deicing salt [4]. The conductivity of the solution was 0.10 ± 0.01 mS cm^−1^, and the chloride concentration was within the freshwater range. Beakers without plants but with solution and plastic discs were used as controls to account for evaporation and for sorption to non-plant surfaces. A small capillary tube feeding pressurized air was placed in each beaker to ensure aeration of the solution. At the end of the experiment, after 5 days (119 h), the remaining solution in each beaker was weighed separately to measure water losses.

The plants were then rinsed in deionized water and dried (45 h at 105 °C) to determine the dry weight of fine roots (<1 mm in diameter), coarse roots (>1 mm in diameter), rhizomes, leaves, stems, and inflorescence.

Samples of 20 mL were taken from each beaker before inserting the plant, 0.5 h after inserting, and after 5 days of treatment. The sampling times were chosen to resemble constant flow through the roots in a stream (0.5 h) and the average hydraulic retention time in a stormwater pond (5 days) [23]. The solution in each beaker was stirred before sampling to ensure a representative sample. The samples were put in 24-mL polyethylene bottles and stored at 3 °C until they were analyzed.

### 2.3. Analysis of Samples

The heavy metal concentrations in the samples were determined using atomic absorption spectrophotometry after filtration through 0.45-µm filters (Sarstedt, Nümbrecht, Germany). A SpectrAA 240, GTA 120 furnace (Agilent, Santa Clara, CA, USA) was used for the analysis of Cd, Cu, and Pb (detection limits 0.006, 0.04, and 0.06 µg L^−1^, respectively). Before the analysis, 5 µL of 65% HNO_3_ mL^−1^ of sample was added; all samples were analyzed with standard additions and 2.2% NaCl solution as modifier to prevent interference from the matrix or variation in chloride concentration that could affect speciation. Blanks consisting of deionized water with the same additions were tested in parallel, so that any traces of metals in the additives could be accounted for. A SpectrAA 55B atomic absorption spectrophotometer (Varian, Palo Alto, CA, USA) with a flame atomizer was used for Zn analysis (detection limit 1 µg L^−1^), using standard additions. Eh was measured with a HI98331 conductivity meter (Hanna Instruments, Johannesburg, South Africa).

### 2.4. Data Analysis

Remaining metal in water after 0.5 and 5 days, compared with the initial concentration, was calculated according to Keizer-Vlek et al. [24] as

Remaining metal in water (%) = (([Me]*_tx_* × V*_tx_*)/([Me]*_t_*_0_ × V*_t_*_0_)) × 100 (1)

where [Me]*_tx_* is the concentration in the water sample at sampling time *tx*, and [Me]*_t_*_0_ is the concentration at the start of the experiment. V*_tx_* and V*_t_*_0_ are the volumes of water in the container at sampling time *tx* and the start, respectively. The changes in volume due to evapotranspiration during the first day were assumed to be negligible.

To enable comparison of species regardless of their size, the net removal per fine root mass per hour was calculated with a method adapted from Sricoth et al. [20] as

Removal rate (µg Me g fine root DW^−1^ h^−1^) = (([Me]_*t*0_ × V_*t*0_ − [Me]b*_tx_* × Vb*_tx_* − [Me]*_tx_* × V*_tx_*)/m_fine root (DW)_)/t (2)

where [Me]b*_tx_* is the concentration in containers without plants at sampling time *tx*, and Vb*_tx_* is the volume in containers without plants at sampling time *tx*; m_fine root (DW)_ is the dry weight of the fine roots, and *t* is the time between the start and end of the period.

Statistical analysis was performed in R version 3.5.1. Comparisons between the removal capacities of plants and non-plant controls on the same sampling occasion were conducted using Anova followed by post hoc analysis using Dunnett’s test. Comparisons between metal concentrations in water at start and at each sampling occasion for each species was made with paired t-tests. Comparisons between removal rates on the same sampling occasion between species were conducted using Anova followed by post hoc analysis using Tukey’s HSD test. In cases in which subgroups of clades and families consisted of at least five species, the average removal rates (per species) were tested against each other using Anova, with *p* < 0.05 used to identify significant differences.

## 3. Results

**Figure 1:**
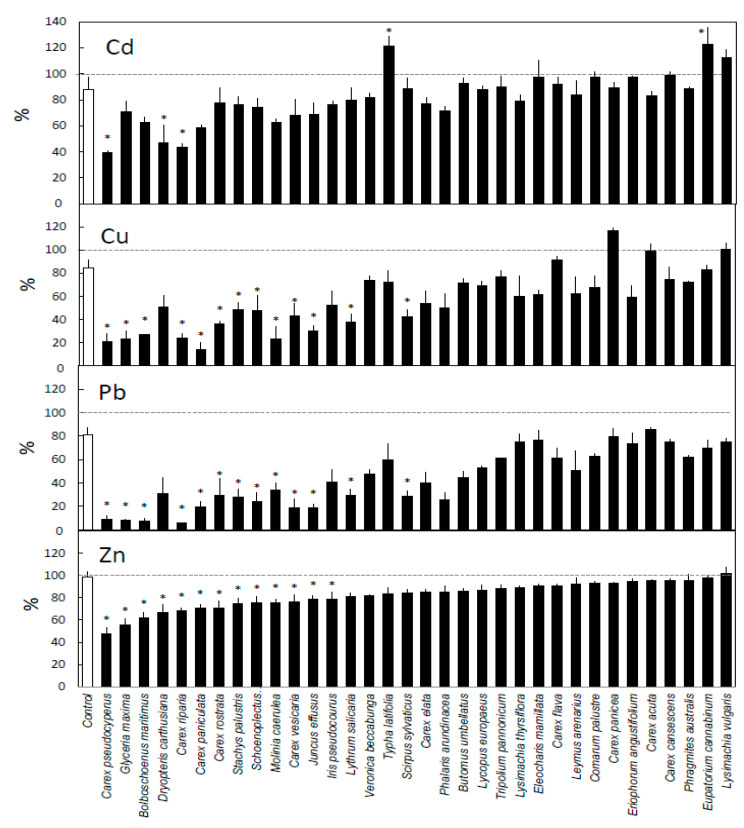
Remaining metal in solution after 0.5 h of exposure. Pairwise comparisons made within each metal between no-plant control (white bar) and every species (black bars), where * = *p* < 0.05. Dotted line represents initial concentration (at 0 h = 100%). *n* = 3, ±SE for all plants, *n* = 21, ±SE, for no-plant control.

**Figure 2:**
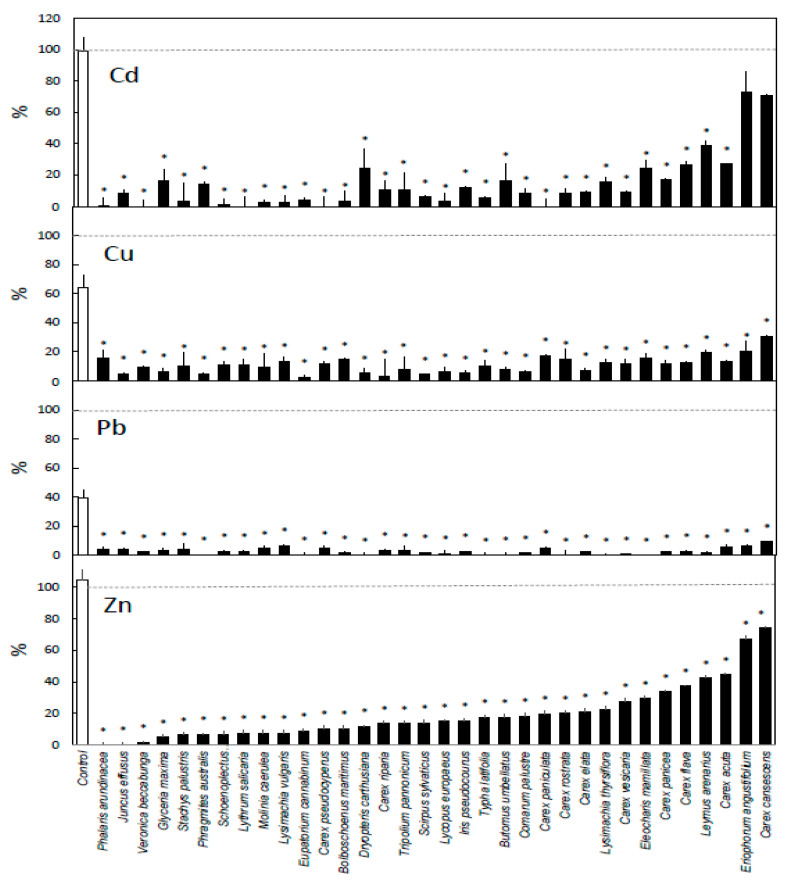
Remaining metal in solution after 5 days of exposure. Pairwise comparisons made within each metal between no-plant control (white bar) and every species (black bars), where * = *p* < 0.05. Dotted line represents initial concentration (at 0 h = 100%). *n* = 3, ±SE for all plants, *n* = 21, ±SE, for no-plant control.

Plants in general decreased the concentration of all tested metals in the water, and only a small share of the metals remained in the water at the end of the experiment for most of the investigated plant species (Figure 1 and Figure 2). The conductivity of the solution remained at 0.1 ± 0.01 mS cm^−1^ throughout the experiment.

After 0.5 h, most plant species had lowered the concentrations of the various metals in the water, compared with the initial concentrations (Figure 1), by up to 61%, 86%, 94%, and 52% for Cd, Cu, Pb, and Zn, respectively. Compared with the beakers without plants, 3, 13, 19, and 13 out of 34 investigated plant species lowered the concentrations of Cd, Cu, Pb, and Zn, respectively. There was large overlap between the species that efficiently removed each metal; all species that removed Cd, Cu, or Zn also removed Pb and usually one or two of the other metals as well. *Carex riparia* and *C. pseudocyperus* stand out, as they significantly decreased the concentrations of all four metals. Fifteen of the species did not remove metal any better than did the beakers without plants after 0.5 h of exposure. In addition, *Typha latifolia* and *Eupatorium cannabinum* increased the concentration of Cd compared with that in beakers without plants and did not remove any of the other metals.

At the end of the test period, after 5 days, sampling showed that all plant species had decreased the concentrations of all four metals compared with both starting concentrations and the beakers without plants (Figure 2). In the best cases, the removal was 100% for Cd, Pb, and Zn and 98% for Cu. On average for all plants, more Cd and Zn than Cu and Pb remained in the water.

The measurements of dried biomass at the end of the experiment indicate large variation between species in both distribution between plant parts and total biomass (Table 1). There was a 38-fold variation in total biomass, with *Dryopteris carthusiana* having the highest and *Eriophorum angustifolium* the lowest. There was a 39-fold variation in fine root mass, with *Glyceria maxima* having the highest and *C. canescens* the lowest. Species with high metal removal generally had high biomass.

The removal rate for the first 0.5 h of exposure was similar for most species (Table 2). A few species stand out. *Butomus umbellatus* and *Lycopus europaeus* had high removal rates for both Pb and Zn, and *Lysimachia thyrsiflora* had high removal rates for Cd and Zn. A number of species released Cd, including *Typha latifolia*, which is commonly used in phytoremediation. The removal rate for the best species was as high as 3.7, 592, 252, 2440 µg g fine root (DW)^−1^ h^−1^ of Cd, Cu, Pb, and Zn, respectively. Unlike the total removal presented above, a species that had a high removal rate for one metal could not be expected to have high removal rates for the other metals as well.

The removal rate for the second period, 0.5 h–5 days, was approximately 100 times slower than during the first 0.5 h (Table 3). The removal rate was up to 0.15, 2.98, 3.58, and 62.9 µg g fine root (DW)^−1^ h^−1^ of Cd, Cu, Pb, and Zn, respectively. The efficient plant species were largely the same for all metals. *Lysimachia thyrsiflora* removed all four metals better than did the species with the lowest removal for each metal.

A few differences in removal rate were found between subgroups of species. Cyperaceae species had a higher removal rate for Zn for the period 0–0.5 h than did other monocot species (*p* = 0.03). Monocots had higher removal rates for Cd and Zn for the period 0.5–5 days than did the eudicots (*p* = 0.01 and *p* = 0.03, respectively).

## 4. Discussion

This work shows that wetland species vary in their ability to remove Cd, Cu, Zn, and Pb from water under identical conditions. Depending on which plant species were present, all or most of the metals added to the water beforehand had been removed after 5 days of exposure (Figure 2). The removal was likely promoted by the lack of nutrients of the solution [25] and slightly demoted by the presence of chloride [26].

### 4.1. General Removal Patterns

Our removal values (Figure 1 and Figure 2) resemble findings of other studies using metal concentrations in the same range [17,27]. The bioconcentration factor values from Weiss et al. [16] after 35 days of exposure to comparable metal concentrations as well as salinity are similar to our removal results. Compared with their results, however, our removal values are slightly higher for Cd, Cu, and Pb, where we had lower metal concentrations in the solution, and lower for Zn, where our concentration was higher. This is in line with studies demonstrating that the bioconcentration factor decreases with increasing concentration [28,29].

The much higher removal rate during the period 0–0.5 h compared with 0.5–5 days (Table 2 and Table 3) is likely because the faster uptake is into the root apoplast during the first half hour, whereas after this period, translocation into the cells and into the rest of the plant will be slower [14]. A similar removal pattern has been observed in other studies [17,30] for all metals examined here.

The remaining metal concentration in the water stabilized at around 5–15% of the initial concentration for many species (Figure 2). This might not necessarily indicate that these plants are unable to remove more metals, just that they are not efficient at these very low concentrations or that the removal is slower than by other plants. A possible example of this is *Schoenoplectus tabernaemontani*. In our study, some of the metals (1%, 11%, 3%, and 7% of Cd, Cu, Pb, and Zn, respectively) remained in the water at the end of the experiment in the *S. tabernaemontani* treatment. In comparison, Weiss et al. [31] found a continuous increase in the metal concentrations in the roots of *S. tabernaemontani* during the first three weeks. Further uptake required root growth, increasing the number of uptake and storage sites. Remaining metal concentrations after longer exposures and at both higher and lower initial concentrations than those used here have also been reported in several other studies [2,17,18,20].

### 4.2. Differences Between Metals

The removal patterns differed between the metals. Lead was generally removed both the fastest and to the greatest extent, closely followed by Cu (Figure 2). This is because Cu and Pb have short retention times in water and readily bind to surfaces such as those of beakers and roots [32]. The removal of Cd and Zn was slower and more remained in the water at the end of the experiment. Additionally, previously accumulated Cd can leak from plant tissues [33], which likely explains the increase in Cd concentration in water for *Typha latifolia* and *Eupatorium cannabium* (Figure 1).

Species that successfully removed one metal after half an hour of exposure also likely removed one or several of the other metals (Figure 1). The only exception found here was for Cd, which, due to its slower removal rate, did not correlate with the removal of other metals. The similarities in removal of combinations of metals corroborated the findings of Deng et al. [19] and Li et al. [15].

### 4.3. Differences Between Species

Large differences between both the species’ ability to remove metal and their removal rates were observed after 0.5 h (Figure 1, Table 2), indicating the potential to increase phytoremediation efficacy by optimal plant choice. The reason for the differences between species likely lies in their morphology [12,14]. Similar variations in metal removal when comparing species under identical conditions were also reported by, for example, Headley and Tanner [2], Ladislas et al. [18], Rai et al. [17], and Weiss et al. [16]. After 5 days of exposure, the plant species differed less in total removal (Figure 2), as most had removed all or nearly all the metal. The removal rate for the period 0.5–5 days instead differed greatly between species, and plants with a high removal rate for one metal also likely had a high removal for other metals (Table 2). It should be noted that since the amount of available metal was limited for all plants due to the low starting concentration, the potential removal rate per unit of biomass decreased as the plant size increased. Consequently, the species with the highest removal rate during the period 0.5–5 days have a small fine root mass and low removal rate during the first half hour, and the species with the lowest removal rate during the period 0.5–5 days have a large fine root mass that removed most of the available metal during the first 0.5 h ( Table 1 and Table 2).

*Carex pseudocyperus* and *C. riparia* stand out from the other species as they quickly reduced the concentrations of all four heavy metals and kept the concentrations low throughout the test. To our knowledge, *C. pseudocyperus* has never been used or studied for phytoremediation purposes. It has high potential to thrive on FTWs, as its root morphology promotes growth and efficient nutrient uptake even under low-oxygen conditions [34]. *Carex riparia* has been evaluated for use on FTWs for remediation of stormwater by Ladislas et al. [12,18], in both greenhouse experiments and field trials. Compared with *Juncus effusus*, which was also included in their study, *C. riparia* accumulated similar amounts of Cd and Zn in greenhouse and field but grew better in field conditions, resulting in higher total removal. Moreover, in the present study, *C. riparia* and *J. effusus* had similar removal rates per unit of biomass, but due to its larger size, C. riparia had a higher total removal (Table 1 and Table 2).

Both *C. pseudocyperus* and *C. riparia* have large biomass (Table 1), mainly consisting of fine roots and leaves. *Carex pseudocyperus* plants lacked rhizomes, and *C. riparia* plants had a higher share of aboveground biomass than did the other species studied here. However, this does not explain why these two species were able to remove Cd after 0.5 h, which *G. maxima* and *C. paniculata* were unable to do, although they had similar biomass values. This indicates that these species could have high-affinity uptake mechanisms for Cd not shared by other plants in the study.

A surprising result of the study is the weak performance of *Phragmites australis* and *T. latifolia*, both popular species in phytoremediation research [7]. This can likely be explained by the composition of their belowground mass, with a low share of fine roots and large rhizomes (Table 1), resulting in low exposure to the pollutants. Their advantages as phytoremediation species depend on their ability to thrive in many climates and their tolerance of high levels of metals [13,35], while stormwater has low levels of metals.

### 4.4. Differences Between Plant Groups

The metal removal rate differed between plant groups in a few cases. Monocot species removed more Cd and Zn after 5 days than did eudicot species, corroborating the results of Li et al. [15]. In contrast, Deng et al. [19] found the opposite, i.e., that eudicots removed Pb and Zn better than did monocots, attributing this to the larger leaves of eudicot species. However, in this study, the monocot species had larger leaf mass than did the eudicots (Table 1), suggesting that metal removal should be predicted directly by leaf size and not by the phylogenetic relationship. On the family level, we found that Cyperaceae species removed more Zn after 0.5 h than did other monocots, contradicting the findings of Li et al. [15], who found them to be among the lowest-accumulating families for Zn. The reason for their high Zn removal in this study could be their frequently large amount of fine roots.

## 5. Conclusions

With this work, we conclude that the ability of wetland plants to remove heavy metals from water differs between species under identical conditions. The species that removed the most metal from water had low removal per unit of biomass but had large biomass consisting mainly of fine roots and leaves. Species that successfully removed one metal also likely removed high amounts of the other metals. Of all investigated species, *Carex pseudocyperus* and *C. riparia* are the most promising candidates for water purification as they could lower the concentration of all four investigated metals after 0.5 h of exposure. The findings of this study should be considered as a starting point for further investigation of plant selection for water purification by using plants for metal removal in treatment wetlands.

## Figures and Tables

**Table 1:** Biomass and origin of species included in the experiment. *n* = 3, ±SE.

Type, Family, Species	Biomass Dry Weight (g)										Origin of Plant Material ^a^
	Fine Roots		Coarse Roots, Rhizomes		Leaves		Stalk, Inflorescence		Total		
**Fern, Dryopteridaceae**											
*Dryopteris carthusiana*	0.71	±0.17	9.22	±0.81	3.13	±0.64	-	-	13.06	±0.19	5
**Monocot, Butomaceae**											
*Butomus umbellatus*	0.08	±0.01	0.74	±0.21	1.16	±0.27	-	-	1.98	±0.15	1
**Cyperaceae**											
*Bolboschoenus maritimus*	0.80	±0.32	1.47	±0.69	0.88	±0.26	0.74	±0.26	3.88	±0.97	2
*Carex acuta*	0.22	±0.05	0.21	±0.15	0.47	±0.15	-	-	0.89	±0.25	3
*Carex cansescens*	0.05	±0.01	0.02	±0.01	0.19	±0.05	-	-	0.26	±0.06	3
*Carex elata*	0.37	±0.04	0.14	±0.03	1.24	±0.19	-	-	1.74	±0.23	1
*Carex flava*	0.17	±0.03	0.10	±0.01	0.58	±0.07	0.14	±0.01	0.91	±0.05	4
*Carex panicea*	0.18	±0.02	0.13	±0.02	1.05	±0.15	-	-	1.36	±0.18	4
*Carex paniculata*	0.51	±0.06	0.58	±0.15	4.31	±0.99	-	-	5.41	±0.16	1
*Carex pseudocyperus*	1.16	±0.21	0.18	±0.08	3.36	±0.12	-	-	4.71	±1.32	5
*Carex riparia*	0.44	±0.03	0.29	±0.05	4.51	±0.15	-	-	5.23	±0.15	3
*Carex rostrata*	0.64	±0.23	0.40	±0.13	1.77	±0.66	0.26	±0.00	2.86	±0.57	5
*Carex vesicaria*	0.46	±0.11	0.23	±0.04	1.86	±0.12	-	-	2.55	±0.16	4
*Eleocharis mamillata*	0.15	±0.12	0.04	±0.02	-	-	0.67	±0.02	0.86	±0.84	5
*Eriophorum angustifolium*	0.10	±0.02	0.06	±0.03	0.18	±0.04	-	-	0.34	±0.04	6
*Schoenoplectus tabernaemontani*	0.40	±0.01	0.51	±0.18	-	-	2.05	±0.36	2.96	±0.16	2
*Scirpus sylvaticus*	0.43	±0.04	0.29	±0.05	1.51	±0.16	-	-	2.23	±0.22	5
**Iridaceae**											
*Iris pseudocourus*	0.97	±0.09	2.41	±0.69	2.64	±0.19	-	-	6.03	±0.80	5
**Juncaceae**											
*Juncus effusus*	0.58	±0.01	0.27	±0.02	2.42	±0.14	-	-	3.27	±0.16	3
**Poaceae**											
*Glyceria maxima*	1.96	±0.31	1.08	±0.24	2.64	±0.70	2.80	±0.84	8.44	±1.67	5
*Leymus arenarius*	0.09	±0.02	0.10	±0.02	0.30	±0.00	0.27	±0.01	0.77	±0.01	2
*Molinia caerulea*	0.70	±0.04	0.23	±0.02	3.01	±0.15	-	-	3.93	±0.16	1
*Phalaris arundinacea*	0.40	±0.06	0.15	±0.01	0.72	±0.15	0.69	±0.09	1.80	±0.41	1
*Phragmites australis*	0.30	±0.06	0.59	±0.10	0.56	±0.06	1.05	±0.19	2.66	±0.21	1
**Typhaceae**											
*Typha latifolia*	0.14	±0.02	0.86	±0.12	1.67	±0.11	-	-	2.67	±0.06	5
**Dicot, Asteraceae**											
*Eupatorium cannabinum*	0.35	±0.03	0.37	±0.15	1.12	±0.28	1.68	±0.36	3.53	±0.08	1
*Tripolium pannonicum*	0.46	±0.08	0.50	±0.07	0.43	±0.12	-	-	1.39	±0.21	1
**Lamiaceae**											
*Lycopus europaeus*	0.09	±0.02	0.08	±0.03	0.61	±0.11	0.27	±0.05	1.04	±0.15	5
*Stachys palustris*	0.30	±0.07	0.27	±0.06	0.85	±0.04	1.14	±0.09	2.47	±0.57	1
**Lythraceae**											
*Lythrum salicaria*	0.25	±0.05	0.56	±0.12	0.39	±0.11	1.43	±0.12	3.24	±0.13	1
**Plantaginaceae**											
*Veronica beccabunga*	0.14	±0.01	0.06	±0.01	0.86	±0.08	0.63	±0.05	1.70	±0.15	5
**Primulaceae**											
*Lysimachia thyrsiflora*	0.08	±0.03	0.69	±0.10	0.95	±0.09	0.51	±0.11	1.68	±0.26	4
*Lysimachia vulgaris*	0.23	±0.02	0.43	±0.03	1.00	±0.11	-	-	1.62	±0.19	5
**Rosaceae**											
*Comarum palustre*	0.14	±0.05	0.41	±0.22	0.37	±0.11	0.13	±0.04	1.06	±0.38	4

^a^ Origin of plant material: 1—Purchased from Vegtech, seeds collected in Sweden; 2—Rådmansö, 59°44′ N 18°56‴ E; 3—Flemingsberg, 59°13‴ N 17°59‴ E; 4—Jumkil, 59°57′ N 17°17‴ E; 5—Norra Djurgården, 59°21′ N 18°04′ E; 6—cultivated from seeds collected at Kristineberg, 65°04′ N 18°44′ E.

**Table 2:** Removal rates during the first 0.5 h of the experiment. Different letters within each metal denote differences among species, where *p* < 0.05. *n* = 3 ±SE.

Species	Removal Rate 0–0.5 h (ug [Me] g DW^−1^ h^−1^)							
	Cd		Cu		Pb		Zn	
*Bolboschoenus maritimus*	1.1 ± 0.2	ab	195 ± 4	a	46 ± 6	ab	1015 ± 17	abc
*Butomus umbellatus*	−0.6 ± 0.5	ab	258 ± 28	a	87 ± 23	ab	1956 ± 107	ab
*Carex acuta*	0.7 ± 0.3	ab	−57 ± 4	a	−26 ± 4	b	459 ± 17	bc
*Carex cansescens*	−4.6 ± 2.1	b	592 ± 161	a	145 ± 126	ab	1534 ± 466	abc
*Carex elata*	0.8 ± 0.4	ab	89 ± 61	a	118 ± 5	ab	384 ± 278	bc
*Carex flava*	−0.5 ± 0.4	ab	−29 ± 28	a	80 ± 27	ab	812 ± 73	abc
*Carex panicea*	0.3 ± 0.2	ab	−146 ± 18	a	4 ± 6	ab	606 ± 180	abc
*Carex paniculata*	1.6 ± 0.7	ab	156 ± 15	a	55 ± 23	ab	312 ± 276	bc
*Carex pseudocyperus*	0.5 ± 1.8	ab	47 ± 22	a	32 ± 59	ab	155 ± 241	bc
*Carex riparia*	1.8 ± 1.8	ab	91 ± 431	a	69 ± 73	ab	282 ± 407	bc
*Carex rostrata*	0.8 ± 0.1	ab	73 ± 13	a	67 ± 12	ab	777 ± 55	abc
*Carex vesicaria*	0.5 ± 2	ab	109 ± 458	a	100 ± 71	ab	712 ± 218	abc
*Comarum palustre*	−1.3 ± 0.1	ab	147 ± 17	a	61 ± 37	ab	1011 ± 160	abc
*Dryopteris carthusiana*	1.4 ± 0.9	ab	47 ± 101	a	6 ± 21	ab	611 ± 606	abc
*Eleocharis mamillata*	0 ± 1.5	ab	147 ± 396	a	60 ± 10	ab	947 ± 775	abc
*Eriophorum angustifolium*	−3.5 ± 0.3	b	592 ± 18	a	1 ± 5	ab	724 ± 37	abc
*Eupatorium cannabinum*	−4.9 ± 0.7	b	−4 ± 70	a	71 ± 28	ab	121 ± 442	bc
*Glyceria maxima*	0.2 ± 0.4	ab	44 ± 14	a	45 ± 22	ab	317 ± 159	bc
*Iris pseudocourus*	0.4 ± 0.6	ab	37 ± 15	a	17 ± 2	ab	242 ± 20	bc
*Juncus effusus*	0.6 ± 1.1	ab	57 ± 28	a	60 ± 37	ab	148 ± 35	bc
*Leymus arenarius*	0.6 ± 0	ab	337 ± 4	a	215 ± 2	ab	736 ± 17	abc
*Lycopus europaeus*	0.3 ± 0.1	ab	245 ± 16	a	252 ± 4	a	1854 ± 83	abc
*Lysimachia thyrsiflora*	−1.0 ± 0	ab	−47 ± 38	a	5 ± 7	ab	4 ± 35	c
*Lysimachia vulgaris*	3.7 ± 0.8	a	467 ± 47	a	8 ± 18	ab	2440 ± 78	a
*Lythrum salicaria*	1.3 ± 0	ab	252 ± 13	a	106 ± 4	ab	1116 ± 51	abc
*Molinia caerulea*	0.5 ± 0.5	ab	125 ± 22	a	41 ± 41	ab	149 ± 157	bc
*Phalaris arundinacea*	1.2 ± 0.3	ab	100 ± 36	a	143 ± 21	ab	404 ± 145	bc
*Phragmites australis*	0.1 ± 0.3	ab	80 ± 28	a	115 ± 25	ab	118 ±25	bc
*Schoenoplectus tabernaemontani*	0.7 ± 0.5	ab	121 ± 36	a	115 ± 42	ab	818 ± 57	abc
*Scirpus sylvaticus*	0.1 ± 0.6	ab	124 ± 54	a	115 ± 22	ab	440 ± 677	bc
*Stachys palustris*	1.2 ± 1	ab	143 ± 65	a	186 ± 27	ab	817 ± 522	abc
*Tripolium pannonicum*	0.3 ± 2.8	ab	29 ± 19	a	32 ± 56	ab	249 ± 72	bc
*Typha latifolia*	−4.9 ± 0.9	b	126 ± 40	a	29 ± 23	ab	1577 ± 73	abc
*Veronica beccabunga*	1.3 ± 0.5	ab	101 ± 20	a	200 ± 3	ab	1120 ± 63	abc

**Table 3:** Removal rates during the period 0.5 h–5 days. Different letters within each metal denotes differences among species, where *p* < 0.05. *n* = 3, ±SE.

Species	Removal Rate 0.5 h–5 days (ug [Me] g DW^−1^ h^−1^)							
	Cd		Cu		Pb		Zn	
*Bolboschoenus maritimus*	0 ± 0	cd	−0.1 ± 0	a	0.3 ± 0	d	7 ± 0	def
*Butomus umbellatus*	0.1 ± 0	ab	3 ± 0.1	a	3.2 ± 0.1	ab	38 ± 0	abc
*Carex acuta*	0 ± 0	bcd	1.8 ± 0	a	1.1 ± 0	abcd	15 ± 0	bcdef
*Carex cansescens*	0.1 ± 0	bcd	2.3 ± 1.3	a	2.9 ± 0.9	abc	28 ± 7	bcdef
*Carex elata*	0 ± 0	cd	0.3 ± 0	a	0.1 ± 0.1	d	7 ± 2	def
*Carex flava*	0.1 ± 0	bcd	1.7 ± 0.3	a	0.8 ± 0.1	bcd	21 ± 3	bcdef
*Carex panicea*	0 ± 0	bcd	1.7 ± 0.7	a	0.9 ± 0.3	bcd	20 ± 2	bcdef
*Carex paniculata*	0 ± 0	cd	−0.3 ± 0	a	0.2 ± 0	d	2 ± 2	ef
*Carex pseudocyperus*	0 ± 0	d	0 ± 0.4	a	−0.1 ± 0.3	d	1 ± 2	f
*Carex riparia*	0 ± 0	cd	0 ± 0.7	a	−0.1 ± 0.3	d	2 ± 2	ef
*Carex rostrata*	0 ± 0	cd	0 ± 0	a	0.1 ± 0	d	6 ± 0	def
*Carex vesicaria*	0 ± 0	cd	0.3 ± 0.8	a	0.1 ± 1.3	d	10 ± 5	bcdef
*Comarum palustre*	0.1 ± 0	bcd	2.2 ± 0.3	a	1.4 ± 0.1	abcd	40 ± 3	ab
*Dryopteris carthusiana*	0 ± 0	cd	0.2 ± 0.5	a	0.3 ± 0.1	d	5 ± 2	def
*Eleocharis mamillata*	0 ± 0	bcd	0.7 ± 1.8	a	1.6 ± 0.9	abcd	24 ± 17	bcdef
*Eriophorum angustifolium*	0 ± 0	bcd	1.1 ± 0.3	a	2.9 ± 0.2	abc	22 ± 5	bcdef
*Eupatorium cannabinum*	0.1 ± 0	bcd	1.6 ± 0.8	a	0.8 ± 0.4	bcd	22 ± 9	bcdef
*Glyceria maxima*	0 ± 0	d	0 ± 0.2	a	−0.1 ± 0.2	d	2 ± 3	ef
*Iris pseudocourus*	0 ± 0	cd	0.2 ± 0.1	a	0.2 ± 0.1	d	4 ± 2	ef
*Juncus effusus*	0 ± 0	cd	0 ± 0.4	a	0 ± 0.3	d	2 ± 2	ef
*Leymus arenarius*	0.1 ± 0	bcd	2.3 ± 0	a	1.7 ± 0	abcd	31 ± 0	bcde
*Lycopus europaeus*	0.1 ± 0	ab	2.7 ± 0.2	a	2.1 ± 0.2	abcd	35 ± 2	abcd
*Lysimachia thyrsiflora*	0.2 ± 0	bcd	1.9 ± 0.1	a	3.6 ± 0	cd	63 ± 0	def
*Lysimachia vulgaris*	0 ± 0	a	1 ± 0.1	a	0.5 ± 0.1	a	8 ± 1	a
*Lythrum salicaria*	0 ± 0	bcd	0.1 ± 0.1	a	0.5 ± 0	cd	20 ± 1	bcdef
*Molinia caerulea*	0 ± 0	cd	−0.1 ± 0.1	a	0 ± 0.1	d	2 ± 1	ef
*Phalaris arundinacea*	0 ± 0	cd	0.1 ± 0.1	a	−0.1 ± 0.1	d		cdef
*Phragmites australis*	0 ± 0	bcd	1.1 ± 0.2	a	0.5 ± 0.2	cd	14 ± 1	bcdef
*Schoenoplectus tabernaemontani*	0 ± 0	cd	0.4 ± 0.1	a	0.2 ± 0	d	12 ± 1	bcdef
*Scirpus sylvaticus*	0 ± 0	cd	0.2 ± 0.6	a	0.1 ± 0.6	d	8 ± 4	def
*Stachys palustris*	0 ± 0	bcd	0.3 ± 0.3	a	0 ± 0.5	d	10 ± 4	cdef
*Tripolium pannonicum*	0 ± 0	cd	0.8 ± 0.7	a	0.4 ± 0.3	cd	8 ± 9	def
*Typha latifolia*	0.1 ± 0	abc	1.4 ± 0.1	a	1.6 ± 0	abcd	26 ± 2	bcdef
*Veronica beccabunga*	0.1 ± 0	bcd	1.3 ± 0.2	a	0.5 ± 0.1	cd	21 ± 2	bcdef

## Supplementary Materials

The following are available online at https://www.mdpi.com/1660-4601/17/13/4623/s1. Table S1: Habitats of the included species.
