# Supplementary material for: Screening the Capacity of 34 Wetland Plant Species to Remove Heavy Metals from Water
Source: Int J Environ Res Public Health. 2020 Jun 27;17(13):4623. doi: 10.3390/ijerph17134623 (PMC7369712; doi:10.3390/ijerph17134623)
Supplement: Supplementary file 1 [file ijerph-17-04623-s001.docx]

**Table S1.** Species included in the experiment with habitat and sampling site; n.d., not defined

| **Species ^a^** | **Type** | **Family** | **Brackish/fresh water ^a^** | **Nutrient demand^a^** | **Origin of plant material^b^** |
| --- | --- | --- | --- | --- | --- |
| *Bolboschoenus maritimus* | Monocot | Cyperaceae | Brackish | n.d. | 2 |
| *Butomus umbellatus* | Monocot | Butomaceae | Fresh | High | 1 |
| *Carex acuta* | Monocot | Cyperaceae | Fresh | Medium | 3 |
| *Carex canescens* | Monocot | Cyperaceae | Fresh | Low | 3 |
| *Carex elata* | Monocot | Cyperaceae | Fresh | Medium–high | 1 |
| *Carex flava* | Monocot | Cyperaceae | Fresh | Low | 4 |
| *Carex panicea* | Monocot | Cyperaceae | Fresh | n.d. | 4 |
| *Carex paniculata* | Monocot | Cyperaceae | Fresh | n.d. | 1 |
| *Carex pseudocyperus* | Monocot | Cyperaceae | Fresh | High | 5 |
| *Carex riparia* | Monocot | Cyperaceae | Fresh | High | 3 |
| *Carex rostrata* | Monocot | Cyperaceae | Fresh | Low | 5 |
| *Carex vesicaria* | Monocot | Cyperaceae | Fresh | Medium | 4 |
| *Comarum palustre* | Eudicot | Rosaceae | Fresh | Low | 4 |
| *Dryopteris carthusiana* | Fern | Dryopteridaceae | Fresh | n.d. | 5 |
| *Eleocharis mamillata* | Monocot | Cyperaceae | Fresh | Low | 5 |
| *Eriophorum angustifolium* | Monocot | Cyperaceae | Fresh | Low | 6 |
| *Eupatorium cannabinum* | Eudicot | Asteraceae | Brackish + fresh | High | 1 |
| *Glyceria maxima* | Monocot | Poaceae | Fresh | High | 5 |
| *Iris pseudacorus* | Monocot | Iridaceae | Brackish + fresh | Medium–high | 5 |
| *Juncus effusus* | Monocot | Juncaceae | Fresh | Low | 3 |
| *Leymus arenarius* | Monocot | Poaceae | Brackish | n.d. | 2 |
| *Lycopus europaeus* | Eudicot | Lamiaceae | Fresh | Medium–high | 5 |
| *Lysimachia thyrsiflora* | Eudicot | Primulaceae | Fresh | n.d. | 4 |
| *Lysimachia vulgaris* | Eudicot | Primulaceae | Fresh | n.d. | 5 |
| *Lythrum salicaria* | Eudicot | Lythraceae | Fresh | Medium | 1 |
| *Molinia caerulea* ssp. *caerulea* | Monocot | Poaceae | Fresh | Low | 1 |
| *Phalaris arundinacea* | Monocot | Poaceae | Brackish + fresh | High | 1 |
| *Phragmites australis* | Monocot | Poaceae | Brackish + fresh | Medium–high | 1 |
| *Schoenoplectus tabernaemontani* | Monocot | Cyperaceae | Brackish | n.d. | 2 |
| *Scirpus sylvaticus* | Monocot | Cyperaceae | Fresh | n.d. | 5 |
| *Stachys palustris* | Eudicot | Lamiaceae | Fresh | Medium | 1 |
| *Tripolium pannonicum* ssp. *vulgare* | Eudicot | Asteraceae | Brackish | n.d. | 1 |
| *Typha latifolia* | Monocot | Typhaceae | Fresh | High | 5 |
| *Veronica beccabunga* | Eudicot | Plantaginaceae | Fresh | High | 5 |
|  |  |  |  |  |  |
| ^a^ Species names, habitats, and nutrient requirements from Mossberg, B.; Stenberg, L. *Nordens flora*; Bonnier Fakta: Stockholm, 2018; ISBN 9789174245264. | | | | | |
| ^b^ Origin of plant material: 1 – Purchased from Vegtech, seeds collected in Sweden; 2 – Rådmansö, 59°44’N 18°56’”E; 3 – Flemingsberg, 59°13’”N 17°59’”E; 4 – Jumkil, 59°57’N 17°17’”E; 5 – Norra Djurgården, 59°21’N 18°04’E; 6 – cultivated from seeds collected at Kristineberg, 65°04’N 18°44’E | | | | | |
